# Stronger brain activation for own baby but similar activation toward babies of own and different ethnicities in parents living in a multicultural environment

**DOI:** 10.1038/s41598-022-15289-1

**Published:** 2022-06-29

**Authors:** Bindiya Lakshmi Raghunath, Kelly Hwee Leng Sng, S. H. Annabel Chen, Vimalan Vijayaragavan, Balázs Gulyás, Peipei Setoh, Gianluca Esposito

**Affiliations:** 1 Psychology Program, School of Social Sciences, Nanyang Technological University, Singapore, Singapore; 2 Department of Psychology and Cognitive Science, University of Trento, Rovereto, Italy; 3 Centre for Research and Development in Learning (CRADLE), Nanyang Technological University, Singapore, Singapore; 4 Lee Kong Chian School of Medicine, Nanyang Technological University, Singapore, Singapore; 5 Office of Educational Research, National Institute of Education, Singapore, Singapore

**Keywords:** Neuroscience, Psychology

## Abstract

Specific facial features in infants automatically elicit attention, affection, and nurturing behaviour of adults, known as the baby schema effect. There is also an innate tendency to categorize people into in-group and out-group members based on salient features such as ethnicity. Societies are becoming increasingly multi-cultural and multi-ethnic, and there are limited investigations into the underlying neural mechanism of the baby schema effect in a multi-ethnic context. Functional magnetic resonance imaging (fMRI) was used to examine parents’ (*N* = 27) neural responses to (a) non-own ethnic in-group and out-group infants, (b) non-own in-group and own infants, and (c) non-own out-group and own infants. Parents showed similar brain activations, regardless of ethnicity and kinship, in regions associated with attention, reward processing, empathy, memory, goal-directed action planning, and social cognition. The same regions were activated to a higher degree when viewing the parents’ own infant. These findings contribute further understanding to the dynamics of baby schema effect in an increasingly interconnected social world.

## Introduction

Do you think babies are cute? When you see a baby in someone’s arms, do you feel warm inside? Now what if this baby does not belong to your ethnic group?.

### Baby-schema effect and its neural basis

Research has shown that compared to faces of adults, faces of infants command greater attention^1–7^. The infant face has a measurable impact upon our perceptions and behaviour^8^—both men and women will expend extra effort to look at cute infant faces for longer^2^. Indeed, the unique and instantly recognizable facial configuration of infants is pleasing and rewarding^2,9^, and an instinctive reaction of adults upon seeing an infant is to smile^10^. The automatic orientation to an infant’s face suggests that it serves as a cue to trigger a distinct set of brain responses that promotes adult adaptive caregiving^11,12^. Importantly, infant facial cues convey salient information that elicit affection and nurturing from adults^13^. This has been termed the baby schema effect^14^.

Several neuroimaging studies have sought to demonstrate the neural bases of the baby schema effect. A study conducted in fathers and non-fathers compared neural processing of infant versus adult faces^15^. This study demonstrated that in fathers, infant faces (compared to adult faces) elicited greater activation in empathy (superior frontal gyrus, precuneus), salience/reward and emotion processing (orbitofrontal cortex, medial frontal gyrus), and mentalizing (temporo-parietal junction, ventromedial prefrontal cortex) networks^15^. Additionally, stronger responses to infant/child faces (versus adult faces) have been reported in visual cortical areas, such as the fusiform gyrus^16–19^, and middle occipital gyrus^16,20^. The right fusiform gyrus in particular is of key importance in face processing^21^ and may play a vital role in encoding baby schema facial features^9,19,22^. These visual cortical areas may serve as an entry point to forward the processed baby face information to other brain regions associated with attention, emotion and memory for further processing and control of behavioural responses^9^. In response to infant facial cues, activation in the premotor/supplementary motor area^16^ and the cingulate^23^ have been identified. These regions have been previously reported in connection with motor intentions and control^24–26^. A common activation reported across disparate studies is in the insula^19,23,27,28^; its response to infant faces has been linked to its role in social-emotional processing, empathy for others, and reward and motivational salience processing (for a review, see Uddin et al.^29^). Meta-analyses of fMRI studies confirmed the role of these neural circuits in parenting^30–34^.

In addition to demonstrating the neural bases of baby schema effect, studies have sought to identify unique parental responses to visual cues in own infant contrasted with non-kin infants. In response to own infant faces (versus non-own infant faces), fMRI studies have found activation of the superior temporal sulcus/gyrus^19,20^, middle temporal gyrus^20,23^, and the fusiform gyrus^19,23^. These regions are all considered part of the empathy network^35–38^, indicative of the importance for parents to accurately understand emotional and social cues from their own infant during interactions, and to appropriately respond to the cues. In addition, studies have highlighted involvement of neural regions associated with reward and parental motivation^32,39–41^, such as the amygdala^19,20,27,28^, the middle occipital gyrus^13,16,20^, and the inferior frontal gyrus^23,42,43^.

It appears from past literature that the baby schema response, regardless of kinship, recruits the empathy, reward and motivation as well as motor intentions and control networks. However, our perception of cuteness in baby schema and subsequent neural responses and behavior are dynamic and may be strongly influenced by psychosocial contexts such as ethnicity^44^. In fact, while multiple studies have found that there is no other-race effect (ORE) for infant faces (e.g., Alley^45^; Chin et al.^46^; Golle et al.^47^; Proverbio & De Gabriele^45–48^)—where ORE is referred to generally as faster reaction time in categorization tasks for one’s ethnic group^49^—investigations have produced mixed results. A recent study has found that regardless of age, ethnic in-group infant faces are better remembered than out-group faces^50^, a finding consistent with the perceptual expertise hypothesis^51^, or the contact hypothesis^52^. Our present study then investigates whether living in a well-integrated multicultural context lends greater expertise to parents toward babies of various ethnicity.

### In-group and out-group perceptions and its neural basis

With rapid globalization and increasing levels of migration^53^, dramatic changes in the racial and ethnic composition of societies worldwide have made social interactions with diverse social groups ubiquitous elements of everyday life^54^. People have an innate tendency to distinguish the “us” from the “them”^55^, activating stereotypes and attitudes that influence our social behaviour accordingly^56–58^. Social perceptions of people as in-group or out-group members, in terms of ethnicity for instance, are perceived effortlessly^59,60^ and are typically conveyed by facial features (e.g., eye shape, jaw’s size, or skin colour).

With regards to ethnic in-group face perception, research focusing on differences in visual encoding have shown greater activations in fusiform and occipital areas for in-group faces when the faces were unfamiliar^61^, thereby suggesting a greater involvement of sensory regions when processing ethnic in-group than out-group stimuli^62,63^. Brain regions involved in empathic processes such as the cingulate cortex^30,64–67^, and insula^68–70^ were associated with in-group stimuli. Striatal responses, associated with reward and approach-related responses^71^, to same-ethnic stimuli have also been reported^68^.

Ethnic out-group face perception is correlated with greater amygdala activity^68^, indicative of enhanced emotional responses to out-group members owing to the link between racial prejudice and affective reactions^72–75^. Regions in conflict monitoring and inhibition of prejudice towards other-race stimuli, specifically the anterior cingulate cortex and dorsolateral prefrontal cortex activity^73,76^, was seen in higher-order cognitive processes involved in race perception. In support of greater visual activity and attentional resources engaged in processing other-group stimuli, activity in the middle occipital gyrus and inferior parietal lobule^68^ have also been reported.

When neural responses of in-group and out-group faces were examined in an integrated multicultural society, perception and processing of faces were dependent on the identification with a common shared culture^77^, suggesting a divergence from the traditional conception of racial categorization. When the society explicitly and positively supports cross-ethnic interactions, an “enlarged in-group” has been reported, such that out-groups are processed similarly to in-groups^77^. In contrast to the classical ethnic in- and out-group categorization, the study by Rigo et al.^77^ demonstrated a form of categorization dependent on culture, and not ethnicity, which shapes and drives spontaneous social judgment of others. This is consistent with an earlier pattern of findings by Zuo and Han^78^, which found no difference in neural activity in the pain matrix for in-group (Asian) and out-group (Caucasian) amongst Chinese adults who grew up in Western countries. Exposure to different cultures in a multi-ethnic society therefore may lead to less out-group-like neural activations.

In summary, while the traditional conception of racial categorization implicates sensory regions, the empathy and reward networks in in-group face perception, versus the recruitment of emotion, self-regulation and attention networks in out-group face perception, research examining a multi-ethnic context as in the present study has suggested a picture where ethnic out-group faces may be processed in a similar manner as an in-group.

### Current study

It is unknown whether in a well-integrated multi-ethnic society the baby schema effect would lead to an equal preference, as evidenced by neural responses, for infant faces regardless of whether the face belongs to an ethnic in-group or ethnic out-group. By investigating brain reactivity and responsiveness to different ethnic infant faces in a multi-ethnic society, we can effectively situate a general model of the social brain in today’s multi-ethnic setting. To the best of our knowledge, no fMRI study has focused on the impact of ethnicity in human parental brain responses to infant facial stimuli through direct comparisons of own infants and ethnic in-group and out-group infants, as this study investigates. As the world is geared increasingly towards multiculturalism, it is imperative to examine the baby schema effect and how parents respond to babies in today’s multi-ethnic context.

Examining baby schema effects in parents in a multi-ethnic context adds ecological validity to the previous work on baby schema that has mostly not considered the effect of psychosocial factors, such as ethnicity in a multicultural setting. Indeed, the growing field of baby schema effect and its implications on parenting needs to continue developing in an increasingly socially and culturally complex and interconnected world^79^. If parents’ neural responsiveness to baby schema is moderated by the infant’s ethnicity, ethnicity might be used to screen parents at risk for showing less optimal caregiving behaviour, or to determine the effectiveness of parenting interventions. Parenting interventions with at-risk dyads could then also focus on increasing the perceived reward value of infant facial cues (i.e., cuteness), which has been found to be modifiable through experience^80^. It is without doubt that understanding how parents’ brains respond to infant faces is vital to optimising parenting, as it is to diagnosing risk, to the new generation of parents who find themselves in today’s multi-ethnic setting.

Given that previous literature has established the neural correlates of baby schema effect as well as racial categorization, we aim to examine if ethnicity plays a role in the baby schema effect in a multicultural society. If classical racial bias exists on the level of infant face perception, we should expect neural activations related to race categorization (as outlined in the literature) regardless of the baby schema effect. However, if racial bias takes the backseat to the baby schema effect, instead of the classical conception of neural correlates of racial categorization, we should expect babies of different ethnicities to elicit activity in the baby schema response network (as outlined in the literature). In other words, we examined the differences in neural responses of parents viewing (1) non-own ethnic in-group infant, and (2) non-own ethnic out-group infant. In addition, neural responses to parents’ (3) own infant are examined. By integrating both ethnicity and kinship, we aim to build a more comprehensive model of the baby schema response network in parents who find themselves in today’s multicultural setting. This study was conducted in Singapore, owing to its well-integrated multi-ethnic setting. It is expected that infant face stimuli of different ethnicity and kinship would induce different brain activation patterns, recruiting different networks, in parents. Taking into consideration the finding of an “enlarged in-group” reported in a multicultural society (Rigo et al.^77^), it is postulated that the baby schema effect is robust in a multi-ethnic context, such that there will be (1) no significant difference in brain activations between viewing non-own ethnic in-group and out-group. In addition, the viewing of one’s own infant (vs. non-own in-group and out-group infants) would be reflected in greater involvement of brain areas underlying (2) empathic processing (insula, fusiform gyrus, superior temporal gyrus, middle temporal gyrus, and inferior temporal gyrus), (3) reward and motivation (amygdala, insula, fusiform gyrus, middle occipital gyrus, middle temporal gyrus, and inferior temporal gyrus), and (4) greater involvement of cerebral regions previously reported in response to own infant faces in connection with motor intentions and control (supplementary motor area, and middle cingulum), which collectively play a pivotal role in parent-infant bonding as the baby schema response network.

## Materials and methods

### Participants

A total of 27 Singaporean Chinese parents (16 females; *M* = 34.91 years, *SD* = 4.93 years) took part in the present study. All participants had at least one child who was 4 years old or younger. Participants were right-handed, with normal or corrected-to-normal vision, and free of neurological and psychological problems. Participants reported not having had travelled overseas over the past 6 months for more than a 2-month period. Participants were requested to abstain from alcohol, caffeine, and nicotine for 24 h before the scan session. All methods were performed in accordance with the relevant guidelines and regulations. This study was approved by the Institute Review Board of Nanyang Technological University (IRB-2017-01-029).

### Experimental task

**Figure 1:**
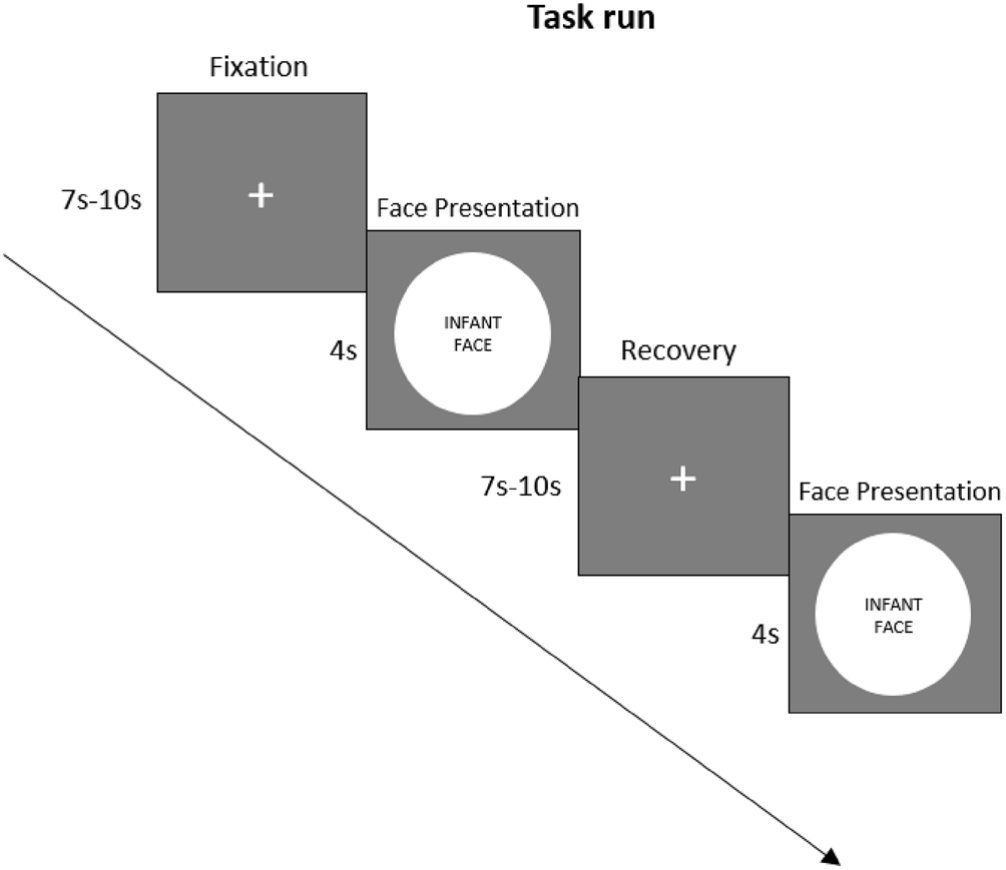
Task paradigm. Every trial began with a fixation cross presented between 7 and 10 s, ensued by 4 s of face presentation of non-own ethnic in-group infant (IG), non-own ethnic out-group infant (OG), or own infant (OI), followed by a recovery period of 7–10 s. All images were presented in random order.

Participants were presented a total of 17 images of faces in an event-related design: (i) 8 Chinese Infant (ethnic in-group; IG), (ii) 8 Indian Infant (ethnic out-group; OG), and (iii) 1 Own Infant (OI) that is repeated 8 times. Except for the image of their own infant, which the participants provided, all face stimuli were sourced from public domain databases^81–83^. Images comprised of black and white, neutrally expressive infant faces (468 × 480 pixels), and were adjusted for brightness and contrast. Each face was within a circle in a grey frame to eliminate the background and minimize potential distraction. In the scanner, participants underwent a session of 24 trials of a duration of 7 to 10 min. The experimental trials began with a fixation cross that was jittered between 7 and 10 s. Every participant saw all images in random order, and each infant face was presented only once, except for participants’ own infant’s face which was presented 8 times due to convenience. Each face was displayed for 4 s (Fig. 1).

To ensure that the infant faces presented can be distinguished by ethnicity, 27 Chinese participants (*M* = 27.04 years, *SD* = 9.32 years), who did not participate in the MRI session, indicated each infant’s race to be Chinese, Indian, Malay, or Other (following the major ethnic groups in Singapore). As faces were correctly classified in terms of in- or out-group, where the in-group accuracy is found to be 97.22% (*SD* = 5.14), and the out-group accuracy is reported at 99.06% (*SD* = 1.75), all faces were included in the analysis.

### Procedure

Participants were briefed about the study and completed informed consent prior the start of the MRI session. Participants also provided informed consent to the use of their own infant faces in the study. All stimuli were presented using the Psychtoolbox package (http://psychtoolbox.org) in MATLAB 2017b and were viewed on a screen in the scanner through a mirror mounted on the head coil. Participants were asked to passively view the presented images. After the experimental session, participants were debriefed and paid S$50 for their participation.

### Imaging data collection

MRI scanning was conducted using a Siemens Magnetom Prisma 3T MRI scanner (Siemens Medical Solutions, Erlangen, Germany) with a 64-channel head-coil. To minimize head movement, participants’ heads were secured for foam padding. Functional scans were obtained with a T2*-weighted gradient echo-planar imaging (EPI) sequence (36 axial slices, interleaved, 3 mm-thickness with no inter-slice gap; TR = 2000 ms; TE = 30 ms; flip angle = 90°; FOV = 192 mm; voxel size = 3 × 3 × 3 mm). A T1-weighted 3D MP-RAGE sequence (192 axial slices, 1 mm-thickness; TR = 2300 ms; TE = 2.25 ms; flip angle = 8°; FOV = 256 mm) was applied to acquire a high-resolution structural scan.

### Data analysis

Imaging data were preprocessed and analysed with SPM12 (http://www.fil.ion.ucl.ac.uk/spm/) on the MATLAB 2019b platform. To allow signal to reach steady-state equilibrium, the first two volumes were discarded. All the remaining volumes were then interpolated in time for slice-timing correction, and realigned to the first volume by rigid-body transformation to offset any effects of head movements. The head motion of all participants was within 3 mm displacement and 2° rotation—no participant was excluded according to the criteria. T1-weighted image was co-registered to the mean functional scan created during realignment, and all functional images were normalised to the T1 MNI-152 template (Montreal Neurological Institute). Finally, the images were smoothed with a 9-mm full-width half-maximum Gaussian kernel. A high-pass filter with a cut-off of 256 s was applied to remove drifts in the signal.

Whole brain analyses were performed. In the first-level individual analysis, general linear modeling (GLM) was conducted for each subject to assess the effect of ethnicity and kinship on the baby schema effect. A total of three conditions: non-own ethnic in-group infant (IG), non-own ethnic out-group infant (OG), and own infant (OI) were modelled as separate regressors of interest. Each regressor was convolved with a canonical haemodynamic response function. Six movement regressors of no interest were also included to model participants’ head movement.

In the second-level mixed-effect analysis, to acquire t-contrast images, specific weight vectors were represented. For the first hypothesis of the study, the contrast of interest was (1.1) IG > OG, to compare the neural differences in viewing non-own ethnic in-group versus out-group infant faces. In addition, a conjunction analysis was performed to investigate the commonalities of neural activation toward non-own ethnic in-group and out-group infant faces, i.e., (1.2) IG ∩ OG. For the second, third and fourth hypotheses, the contrasts of interest were (2.1) OI > IG, to compare the neural differences in viewing own infant versus non-own ethnic in-group infant faces; and (2.2) OI > OG, to compare the neural differences in viewing own infant versus non-own ethnic out-group infant faces. In the second-level random-effect analysis for group effects, a one-sample *t*-tests were performed.

Only clusters that survived voxel-level correction, at a statistical threshold of *p* < 0.05 (*k* = 10 voxels), for multiple comparisons across the whole brain (family-wise error, FWE), were reported.

## Results

### Effect of ethnicity on the baby schema effect

#### IG > OG contrast

To investigate the neural differences between viewing non-own ethnic in-group versus non-own ethnic out-group infant faces, the contrast IG > OG was assessed. No significant difference was found in brain responses to non-own ethnic in-group and out-group infant faces.

#### IG ∩ OG contrast

**Figure 2:**
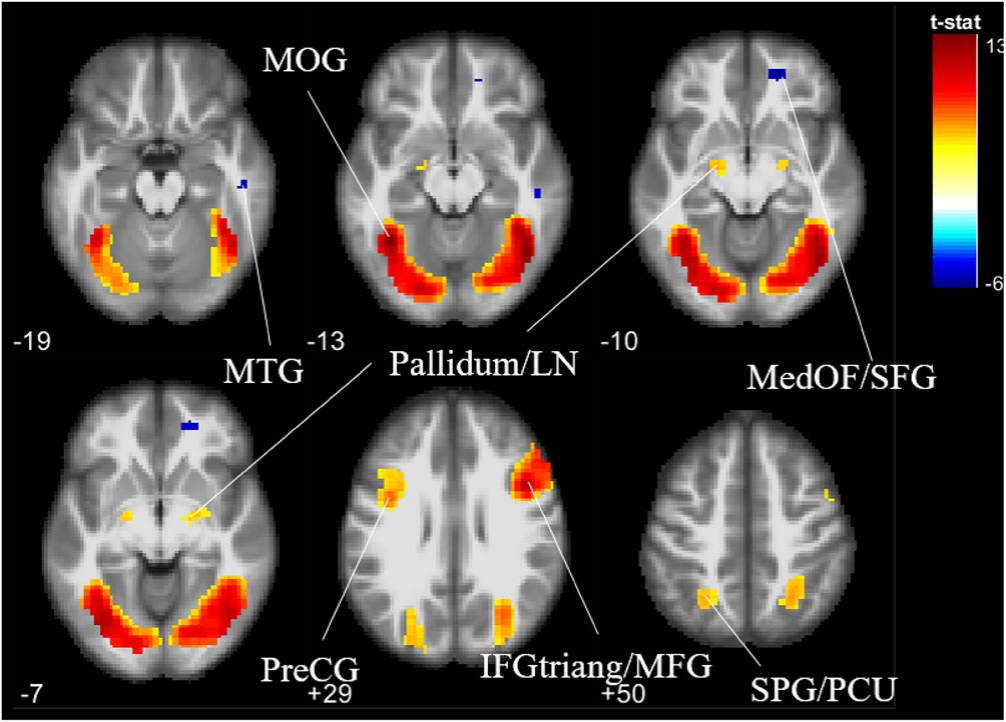
Similarities in brain activations and deactivations to non-own ethnic in-group versus non-own ethnic out-group infant faces (IG ∩ OG), p < 0.05 (FWE corrected). *MOG* middle occipital gyrus, *MTG* middle temporal gyrus, *LN* lentiform nucleus, *MedOF* medial orbitofrontal gyrus, *SFG* superior frontal gyrus, *PreCG* precentral gyrus, *IFGtriang* pars triangularis of the inferior frontal gyrus, *MFG* middle frontal gyrus, *SPG* superior parietal gyrus, *PCU* precuneus.

To examine the similarities in neural responses when non-own ethnic in-group and non-own ethnic out-group infant faces were viewed, a conjunction analysis was performed, and the contrast IG ∩ OG was assessed. For both non-own ethnic in-group and out-group faces, the regions significantly active were middle occipital gyrus, precentral gyrus, and superior parietal gyrus comprising of the precuneus in the left hemisphere; pars triangularis extending to the middle frontal gyrus in the right hemisphere; and bilateral pallidum comprising of the lentiform nucleus. Similarity in deactivation was found in the medial orbitofrontal gyrus extending to superior frontal gyrus, and in the middle temporal gyrus in the right hemisphere (see Fig. 2).

### Effect of kinship on the baby schema effect

#### OI > IG contrast

**Figure 3:**
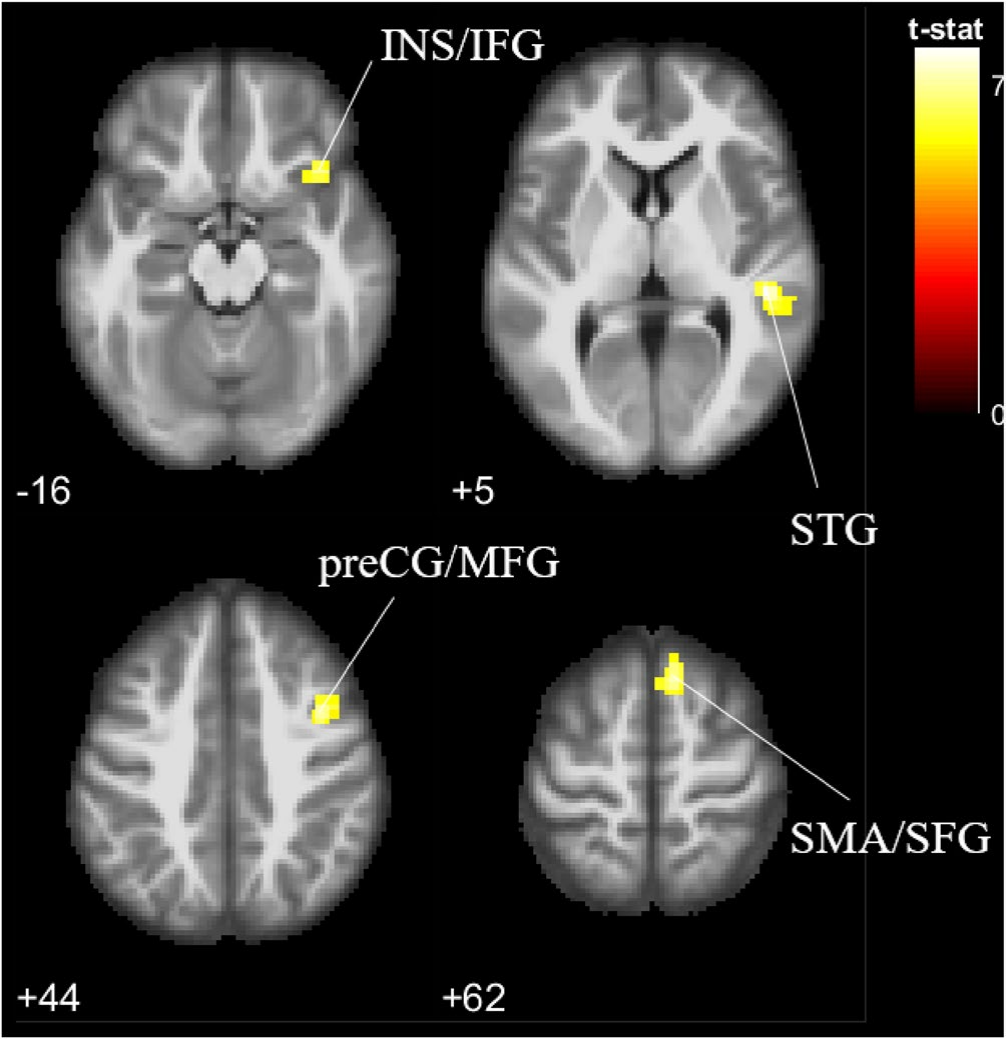
Brain activation to own infant versus non-own ethnic in-group infant faces (OI > IG), p < 0.05 (FWE corrected). *INS* insula, *IFG* inferior frontal gyrus, *STG* superior temporal gyrus, *preCG* precentral gyrus, *MFG* middle frontal gyrus, *SMA* supplementary motor area, *SFG* superior frontal gyrus.

To investigate the differences in neural responses of viewing own infant to non-own ethnic in-group infant faces, the contrast OI > IG was assessed (Fig. 3). Chinese parents showed higher activation in the superior temporal gyrus; supplementary motor area located in the superior frontal gyrus; precentral gyrus extending to the middle frontal gyrus; and insula extending to the inferior frontal gyrus. All activations were limited to the right hemisphere. Deactivation was found in the right parietal sub-gyral and precentral gyrus at uncorrected p < 0.001 (not shown here).

#### OI > OG contrast

**Figure 4:**
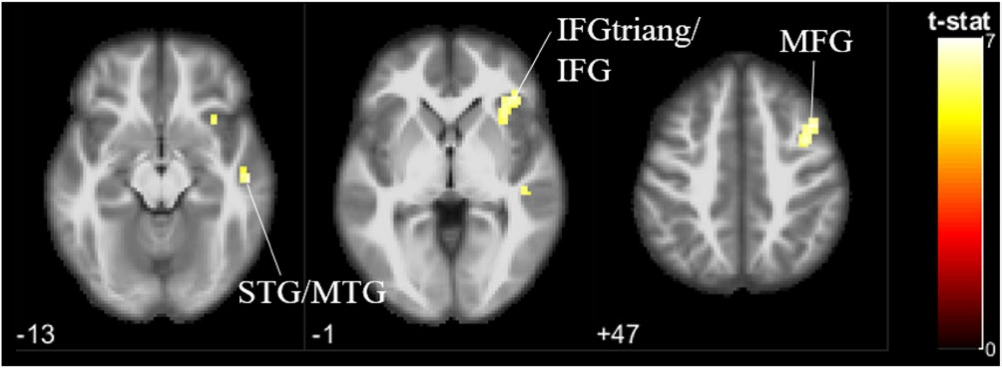
Brain activation to own infant versus non-own ethnic out-group infant faces (OI > OG), p < 0.05 (FWE corrected). *STG* superior temporal gyrus, *MTG* middle temporal gyrus, *IFGtriang* pars triangularis of the inferior frontal gyrus, *IFG* inferior frontal gyrus, *MFG* middle frontal gyrus.

To investigate the differences in neural responses of viewing own infant to non-own ethnic out-group infant faces, the contrast OI > OG was assessed (Fig. 4). Chinese parents showed higher activation in the middle frontal gyrus; pars triangularis in the inferior frontal gyrus; and superior temporal gyrus extending to the middle temporal gyrus. Similar to the OI > IG contrast, activations were all restricted to the right hemisphere. Only at the less stringent uncorrected p < 0.001, deactivation was found in the bilateral postcentral gyrus, and left precuneus (not shown here).

The results are shown in Table 1.

**Table 1:** Peak MNI coordinates of whole-brain activations from the SPM contrasts (p < 0.05 with FWE correction at the voxel level).

Anatomical region	Cluster size	t-value	Peak MNI		
			X	Y	Z
**Contrast: IG > OG**					
None					
Contrast: OG > IG					
None					
**Contrast: IG ∩ OG (activation)**					
Left middle occipital gyrus	2873	7.66	− 39	− 55	− 13
Right inferior frontal gyrus (pars triangularis)/middle frontal gyrus	505	6.95	45	11	29
Left precentral gyrus	128	5.66	− 39	2	29
Left pallidum/lentiform nucleus	19	5.54	− 18	− 10	10
Left superior parietal gyrus/precuneus	67	5.47	− 27	− 58	50
Right pallidum/lentiform nucleus	23	5.30	18	− 10	− 7
**Contrast: IG ∩ OG (deactivation)**					
Left temporal sub-gyral	27	5.59	− 39	− 43	2
Right posterior cingulate	17	5.55	21	− 46	23
Right medial orbitofrontal gyrus/superior frontal gyrus	13	5.41	18	50	− 10
Right middle temporal gyrus	13	5.46	51	− 22	− 19
Right temporal sub-gyral	16	5.26	36	− 40	− 1
**Contrast: OI > IG**					
Right superior temporal gyrus	92	6.23	48	− 31	5
Right supplementary motor area/superior frontal gyrus	46	6.27	9	20	62
Right precentral gyrus/middle frontal gyrus	40	6.18	39	5	44
Right insula/inferior frontal gyrus	82	6.08	39	20	− 16
Contrast: IG > OI*					
Right parietal sub-gyral	4	3.64	33	− 43	26
Right precentral gyrus	2	3.53	36	− 19	68
**Contrast: OI > OG**					
Right middle frontal gyrus	49	6.15	42	14	47
Right inferior frontal gyrus (pars triangularis)/inferior frontal gyrus	145	6.11	39	32	− 1
Right superior temporal gyrus/middle temporal gyrus	30	6.10	54	− 19	− 13
Contrast: OG > OI*					
Right postcentral gyrus	18	3.67	18	− 43	59

Results of contrasts marked with asterisk (*) are reported at p < 0.001 (uncorrected).

## Discussion

Babies motivate caregiving behavior through their baby schema facial features. Yet research on racial bias suggests that individuals have a propensity to favour their ethnic in-group members over out-group members. The main purpose of the present study was to investigate the effect of in-group and out-group baby schema in a multi-ethnic context. By examining the differences in neural responses toward viewing non-own ethnic in-group and out-group infants, the study sought to build a more integrated image of the parental brain in a multi-ethnic society. The baby schema effect showed robustness in the context of ethnicity, such that there was no difference in brain activations between viewing a non-own ethnic in-group infant and a non-own ethnic out-group infant. Activations were found in networks including the empathy, reward, and motor networks. In addition, own infant faces (vs. non-own ethnic in-group and out-group infants) were found to induce stronger brain activation patterns in these networks.

In line with the first hypothesis that postulated baby schema effect is robust in a multi-ethnic context, Singaporean Chinese parents showed similar brain activations when viewing pictures of babies, regardless of the babies’ ethnicity, in regions associated with attention, reward processing, empathy, memory, goal-directed action planning, and social cognition.

The activations observed in the bilateral pallidum (located in the basal ganglia) and left middle occipital gyrus may be related to reward and motivation processing when viewing non-own infant faces. The pallidum region is implicated in reward and motivation processing^84–86^. Previous studies^87,88^ have similarly reported an activation in the basal ganglia upon viewing infant facial cues. Given the salience and motivational potential of social stimuli, it has been hypothesized that processing reward of same-group stimuli could motivate social behavior^89^. However, it appears that this in-group effect did not apply to infant faces. Our results show that regardless of ethnicity, infant faces alike present rewarding properties that elicit similar approach-related responses. Although the middle occipital gyrus is implicated in initial face perception and processing^90^, it forms connections to specialized cortices involved in emotional and value processing^40^. The left middle occipital gyrus is reported to also impinge on different aspects of categorization and evaluation of socially relevant stimuli^91^, such that racial perception has been found to be represented in this cortical area. Findings of this study demonstrate the effect of baby schema and absence of race bias, such that differences in race of the infant face stimuli do not produce differential activation in the left middle occipital gyrus.

Similar visual activity and attentional resources may have been engaged during processing of non-own infant stimuli regardless of ethnicity. Although a socio-functional framework suggests the strategic benefit of allocating attention towards unfamiliar stimuli^92–94^, and therefore other-group members should capture early attention more than same-group members^60,95,96^, the current study found the absence of race bias in attention when viewing infants of different ethnicity. Interestingly, the activation in the superior parietal lobule comprises of the precuneus in the right hemisphere. The precuneus is implicated not only in attention^9,17,97^, but also empathy^98–100^, and episodic memory retrieval^101^. Participants, being parents in a multicultural context, may be highly experienced with infant faces of various ethnicity, and therefore find them highly personally relevant and easy to identify with and to recognise. The multicultural environment may have enabled a “parental instinct” that motivates caregiving of babies of other ethnicity. Indeed, common activation of the precuneus in parents responding to own child stimuli across visual and auditory stimuli have been reported (e.g., Leibenluft et al.^17^). The present study adds to extant literature by including the activation as a response to non-kin, out-group ethnic infants.

The pars triangularis activation is implicated in face processing at a higher level^102^, action observation and social cognition^103^. The activation of pars triangularis in this study in particular, may demonstrate an imagination of performing goal-directed movement sequences^104,105^. The similar response in the pars triangularis when viewing non-own in-group and out-group infants in parents may reflect a tagging of the infant stimuli as the current focus, which promotes further processing in parental brain networks^13^ (Zhang et al.). Interestingly, activation in the pars triangularis was found to extend to the middle frontal gyrus in the right hemisphere. The middle frontal gyrus is likewise implicated in face processing^15^ (Mascaro et al.), and the right side in particular has been found to act as a gateway between top-down and bottom-up control of attention^106^. Both non-own ethnic in-group and out-group infants may therefore demand equally high levels of orienting and processing from adult caregivers. The middle frontal gyrus has been implicated in adult fMRI studies of face processing in general^107^ and own- and other-race face processing in specific^72,74^. Findings from this study broaden the literature to include its implications in face processing of infants that are of the same or different ethnicity as the perceiver.

Overall, the lack of difference in neural responses to non-own ethnic in-group infant faces and non-own ethnic out-group infant faces suggests that ethnic in-group and out-group are processed similarly with regards to infant faces, especially in terms of attention, reward processing, empathy, memory, goal-directed action planning, and social cognition. This largely supports claims that the infant face elicits similar attention from adults regardless of ethnicity^5^. Indeed, the simple facial configuration of the baby schema has been argued to trigger caregiving motivation independent of ethnicity^8,9,45^. However, we have also found possible neural links to similar levels of memory and cognition toward babies regardless of their ethnicity. This contrasts extant findings that have purported same-ethnicity faces are better recalled than other-ethnicity faces, regardless of age (e.g., Meissner and Brigham^108^), leadings to some authors to argue that the ORE exists for infant faces^50^. Previous findings are possibly due to their participants’ lack of contact with infants^51^, and those of various ethnicity^109^. Our current pool of participants are parents, who hold a higher level of perceptual expertise of viewing infant faces than what are generally sampled (e.g., non-parent college students in Martinez et al.^50^), and the multicultural context may have resulted in an unconscious categorization of ethnic out-group infant faces into an “enlarged in-group” which results in similar preference toward in- and out-group faces. Our suggestion is supported by findings that infant faces are better distinguished among individuals who have experience with infants, such as maternity-ward nurses^110^.

In addition, we expected greater activation of neural regions which play a pivotal role in parent-infant bonding, particularly regions involved in empathic processing, reward and motivation, and motor intentions, when viewing own-infants compared to non-own infants (ethnic in-group and out-group).

As expected (second hypothesis), neural activations for empathic processing in response to own infant face (versus non-own ethnic in-group and out-group infant faces) was evident. Parents showed greater activation in the right insula and right superior temporal gyrus, which are involved in emotional empathy^35,36,38,111^. Additionally, the right inferior frontal gyrus has been proposed to be a key anatomical structure associated with emotional empathy^43^, and its activation is associated with specific components of emotional recognition^112,113^. Empathy is an important contributor to successful social interaction and allows for the prediction and understanding of another’s behavior, and for recognizing and reacting accordingly to infants’ needs^13^. The greater response of these brain regions to own infant face may reflect higher empathic responsiveness toward own babies in parents, which likely plays a critical role in facilitating caregiving behavior toward the infant^114^. Parents need to be able to respond appropriately to their own babies’ cues like crying or smiling^115^, which would enable the detection and interpretation of their child’s signals—an essential prerequisite for sensitive parenting behavior^116^. Functioning of the empathy network also allows parents to respond to infant pain and emotion by representing it in themselves^69^. Therefore, parents’ empathic responsiveness to the baby schema features of their own baby could be interpreted as a first manifestation and a proxy for general responsiveness to child cues.

Results corroborated with the third hypotheses that there would be greater brain activation associated with reward and motivation areas in response to own infant face (versus non-own ethnic in-group and out-group infant faces). Parents showed greater activation in the right insula, right middle temporal gyrus, and right inferior frontal gyrus for own infant faces compared to non-own infant faces. These regions are implicated in reward and motivation processing. Previous research with parents also found increased insular activity particularly to faces of parents’ own infants, resulting in the suggestion that the insula is important for parental caretaking motivation^15,17,23,27,28,39,117^. Similar activations have also been previously found in the inferior frontal gyrus^23,42,43^. The middle temporal gyrus plays a role in reward processing (e.g. Murray et al.^118^) and infant face stimuli^16,119^, but there is little to no evidence in existing literature showing the activation of the middle temporal gyrus as a reward and motivation processing network for own infant faces. This study is the first of its kind to report such an activation.

Finally, consistent with the fourth prediction of greater activation in the motor intentions and control regions for own infants compared to non-own infants, activation was found in the right supplementary motor area. The supplementary motor area located in the superior frontal gyrus has been reported to be implicated in the preparation for movement and the conscious intention to move^24,25^, imagining to grasp^120^, and experiencing an “urge” to move^121^. In fact, the superior frontal gyrus plays a role in visually guided movements^122^, and is associated with the generation and control of relevant motor action^119^. The supplementary motor area is also implicated in the empathy network^69^. Its activation has been similarly reported in studies (e.g., Caria et al.^16^) investigating viewing of infant facial cues. Activation of motor-related regions in response to own infant faces suggests preparation by parents for communicative behavior with infants as well as attachment and caregiving^43^. As described by Lorenz^14^, this forms the biological basis for the “innate releasing mechanism” for affection and nurturance.

The engaged brain networks may be seen in context of the development of three main “parental capacities,” which apply to all caregivers^123,124^. The first parental capacity is emotional scaffolding, which is the ability to empathize and perceive changes in emotion and stress in the infant and support them to regulate their emotions, especially when the infant is distressed. The second parental capacity is the ability and motivation to focus attention on the infant’s emotional cues and respond contingently and responsively, which predicts later cognitive development^125^. The third key parental capacity is the sensitivity to an infant’s attachment behaviors, and to respond appropriately. Here the results demonstrate the brain networks involved in parents in perceiving their own infant’s facial cues (activated to a higher degree than non-own infants), which are essential for the ability to perceive emotional state (empathy), hence provide emotional scaffolding when necessary during instances such as crying^126^, and to hone sensitivity to an infant’s attachment behaviors (reward and motivation), allowing for appropriate responding (motor intentions and control). Such activity is theorized to comprise a “caregiving instinct” that may prepare the individual to provide care to one’s infant by coordinating responsiveness and readiness for sociality.

In line with the literature (e.g., Zhang et al.^13^), parents when viewing their own infant compared to non-own infants also showed higher activation in the theory of mind areas (i.e., middle frontal gyrus). Theory of mind, the ability to understand the mental state of others that underlies behavior, crucially contributes to human social cognition and parenting^127^. Theory of mind network (medial prefrontal gyrus, temporoparietal junction, precuneus) supports the parent’s capacity to read their infant’s non-verbal signals and infer the infant’s intentions^127^. Studies suggest that the transition to parenthood induced in parents changes in brain regions which partially overlap the theory of mind network (e.g., Hoekzema et al.^128^), and that parenthood modulates human brain functions and enhances social cognition in response to infant cues^111,129,130^. It is important to note here that although this theory of mind region showed greater activation towards own-infants, parents viewing non-own infants regardless of their ethnicity also showed middle frontal gyrus activation.

Findings from the present study demonstrate that own babies engage brain regions involved in especially empathic processing, reward and motivation, and motor intentions and control to a higher degree than babies who are not one’s own. These regions are critical for parenting and in ensuring the establishment of a secure parent-infant attachment for future proper child development. However, an important finding is that although not to the extent of an own-baby, non-own babies regardless of ethnicity, also elicit brain activations associated with empathic processing, reward and motivation, and motor intentions and control in parents who find themselves in a multicultural environment. In addition, regions associated with attention and memory are also implicated. However, as our present study did not perform any localizer task that can isolate regions related to a specific cognitive function, we are hesitant to relate neural activity found to certain cognitive processes. Furthermore, in the same experimental session but separate runs, we presented participants with adult faces as well. Though unlikely, we cannot rule out the possibility that adult faces may have had confounding effects on the response to infant faces. In addition, future studies should directly compare participants with (i.e., parents) and without (i.e., non-parents) experience with infants, and participants from cultures that are less multi-ethnic to better establish whether perceptual experience is key to explain present findings. In addition, we only examined Chinese participants (the majority ethnic group in Singapore), future studies need to examine whether minority ethnic groups also find similar effects. Extant literature on ORE mainly samples one ethnicity (e.g., Ackerman et al.^131^; Proverbio et al.^5^), and when they include multiple ethnicity, the environment was not so well-integrated multi-culturally (e.g., White adults with little experience with Black children in Martinez et al.^50^). It is important for future studies to be conducted in a well-integrated multicultural setting, and to sample various ethnic groups.

## Conclusion

The present study indicates that in a multicultural environment, babies regardless of ethnicity induce activations that promote adaptive caregiving. In a multicultural setting, parents may find facial cues of infants regardless of their ethnicity to provide privileged access to neural mechanisms that ignite motivational states across the whole brain, a phenomenon thought to be self-supporting and “metastable” in nature^8^.

## Data Availability

All the data are available at: 10.21979/N9/H0CPDV.
